# Exploring the role of race and ethnicity in resident-to-resident aggression: a qualitative study

**DOI:** 10.1093/geront/gnag209

**Published:** 2026-09-12

**Authors:** E-Shien Chang, Hannah Çongar Mason, Michelle Ma, Taimur Mirza, Jeanne Teresi, Charles Mouton, Danya Keene, Sonya Jhaveri, Sara J Czaja, Mark Lachs, Tony Rosen, Karl Pillemer

**Affiliations:** Division of Geriatrics and Palliative Medicine, Weill Cornell Medicine, New York, New York, United States; Division of Geriatrics and Palliative Medicine, Weill Cornell Medicine, New York, New York, United States; Department of Emergency Medicine, Weill Cornell Medicine, New York, New York, United States; Division of Geriatrics and Palliative Medicine, Weill Cornell Medicine, New York, New York, United States; ArchCare: Continuing Care Community of the Archdiocese of New York, New York, New York, United States; Columbia University Stroud Center, New York State Psychiatric Institute, New York, New York, United States; Department of Family Medicine, University of Texas Medical Branch, Galveston, Texas, United States; Department of Social and Behavioral Sciences, Yale School of Public Health, New Haven, Connecticut, United States; Division of Geriatrics and Palliative Medicine, Weill Cornell Medicine, New York, New York, United States; Division of Geriatrics and Palliative Medicine, Weill Cornell Medicine, New York, New York, United States; Division of Geriatrics and Palliative Medicine, Weill Cornell Medicine, New York, New York, United States; Department of Emergency Medicine, Weill Cornell Medicine, New York, New York, United States; College of Human Ecology, Cornell University, Ithaca, New York, United States

**Keywords:** Long-term care, Elder mistreatment, Health disparities

## Abstract

**Background and Objectives:**

Resident-to-resident aggression (RRA) is the most common form of elder mistreatment in long-term care settings. Although race/ethnicity is likely a contributing factor, research has been limited in examining the role of residents’ race and ethnicity in RRA. We sought to define and qualitatively examine the predisposing factors and acute precipitants of race/ethnicity-related resident-to-resident aggression (RE-RRA).

**Research Design and Methods:**

Guided by the socioecological model, events-based perspective, and conceptual work on stigma-based aggression, we developed qualitative interview and data analytical guides. We conducted nine focus groups and twelve in-depth interviews with 81 nursing home staff from multiple clinical and non-clinical roles across two long-term care facilities on the U.S. East Coast. We coded qualitative data using a deductive thematic qualitative approach.

**Results:**

RE-RRA appeared to be a recurring interpersonal dynamic, though its full scope is likely under-detected and under-recognized. We identified five major types of RE-RRA, including physical violence, verbal harassment, racial/ethnic stereotyping, microaggression, and social exclusion. Ten predisposing factors and eight acute precipitants uniquely contributed to RE-RRA, highlighting the importance of considering distinct race- and ethnicity-related elements in prevention research. We summarized our findings in a conceptual framework for RE-RRA that may be useful for future research.

**Discussion and Implications:**

RE-RRA is a distinct phenomenon shaped by identifiable predisposing and precipitating factors, offering opportunities for future prevention and intervention development. These findings underscore the importance of integrating RE-RRA within staff education, existing clinical governance structures, including incident reporting, and quality improvement processes.

## Background and objectives

Resident-to-resident aggression (RRA) is defined as negative and aggressive physical, sexual, or verbal interactions between long-term care (LTC) residents that in a community setting would likely be construed as unwelcome and have high potential to cause physical or psychological distress in the recipient (Lachs et al., 2016). RRA occurs commonly in LTC settings, with an estimated of 20.2% of residents involved in incidents over a one-month period. (Lachs et al., 2016; Pillemer et al., 2022). RRA is associated with an increased risk of physical and psychological harm to residents (DeBois et al., 2020; Shinoda-Tagawa et al., 2004; Trompetter et al., 2011; Woolford et al., 2021). Exposure to RRA also increases occupational hazards for staff members, adding further strain to an already overstretched workforce (Jain et al., 2018; Yan et al., 2025). Despite its frequency and impact, RRA remains understudied, leaving gaps in prevention, policy, and staff training (National Academies of Science Engineering and Medicine, 2022).

As LTC communities in the U.S. become more racially and ethnically diverse, it is essential to consider the role of race and ethnicity in RRA. Between 1999 and 2008, the number of older Hispanic and Black nursing home (NH) residents grew by 54.9% and 10.8%, respectively, while the number of White residents declined by 10.2% (Feng et al., 2011). Greater racial/ethnic diversity in the resident population increases the likelihood of daily close interactions between residents from diverse backgrounds, many of whom may have had limited prior exposure to racial/ethnic out-groups. Disinhibited behaviors common in cognitive impairment may lead residents without realizing their impact to exhibit racially biased behaviors that would have been suppressed before the loss of executive function (Miller et al., 1997). Indeed, broader patterns of racism embedded in contemporary U.S. society may manifest within LTC facilities through everyday interactions (Shippee et al., 2022). Additionally, the LTC workforce is also among the most racially and ethnically diverse in the healthcare sector (Osterman, 2017). The convergence of resident and workforce diversity may pose additional challenges related to staff-resident communication, conflict mitigation, and potential bias that increases the risk of RRA related to racial/ethnic differences (Chang et al., 2024).

Emerging research has begun to investigate whether differences in residents’ racial and ethnic identities may relate to varying levels of risk for involvement in RRA episodes. Qualitative studies identified episodes in which residents directed racial or ethnic epithets at one another, with such incidents frequently triggering episodes of aggression (Rosen et al., 2008). Another large cohort-based study found that incidents of RRA were less common among Black residents, relative to their White counterparts (Pillemer et al., 2022). A possible explanation was that Black individuals are more likely to receive increased scrutiny when expressing anger, potentially shaping how RRA is perceived and documented (Pillemer et al., 2022). A recent mixed-method study further identified notable differences between residents involved in overt racial and ethnic conflicts and those engaged in other forms of RRA (Chang et al., 2024). This finding aligns with the broader interpersonal violence literature that suggests racially and ethnically-motivated aggression may operate through different mechanisms and require tailored prevention and intervention strategies (Earnshaw et al., 2018).

Notably, an evidence-based RRA intervention has been implemented in the U.S. nursing homes and assisted living facilities, demonstrating a positive impact on staff knowledge and reporting (Teresi et al., 2013, 2026), as well as reducing resident falls and injuries (Teresi et al., 2018). However, the existing intervention does not address the role of race and ethnicity in RRA. Addressing race and ethnicity in LTC staff training on RRA would allow the development of prevention and intervention strategies to identify and mitigate these incidents. Filling this gap will also contribute to better preparation of the LTC workforce to care for the increasingly diverse population of residents with cognitive impairment (Berlowitz et al., 2003).

However, to date, the role of race and ethnicity in RRA remains significantly underexplored due, in part, to challenges studying the phenomenon. Incidents of RRA are typically ephemeral, occur in private spaces that are out of sight of LTC staff, and often take place between residents with cognitive impairment who are unable to reliably report on the incident. Additionally, RRA may be frequently tolerated and presumed to be an accepted part of life in LTC settings (Hall et al., 2009; Jain et al., 2018). This tolerance may be resulted from ageism (Chang et al., 2020) and the dehumanization of older adults with cognitive impairment (Werner, 2014). Despite these challenges, there is an urgent need for more systematic research in this area.

### Defining race and ethnicity-related resident-to-resident aggression

We define race and ethnicity-related RRA as *resident-to-resident aggression motivated by racial or ethnic differences, or conflicts that would not have occurred between residents of the same race or ethnicity*. In such episodes, residents may be targeted because of their racial/ethnic identities and associated characteristics. These episodes may include those with or without explicit racial/ethnic conflicts. Additionally, these episodes likely reflect deeper social dynamics within care environments, posing risks to residents’ safety and further creating divisions within communal living spaces.

### Conceptual perspectives

Our approach to examining the role of race and ethnicity in RRA and developing a tailored framework for future intervention programming is informed by the socioecological model, an events-based perspective, and conceptual work on stigma-based aggression. The socioecological model is well-suited to study RRA, given RRA arises from the interplay of individual, relational, organizational, and societal factors, making both its causes and consequences multifaceted (Burnes et al., 2021; Ferrah et al., 2015). This perspective is particularly important for the study of race and ethnicity in RRA because the effects of residents’ racial and ethnic backgrounds are shaped through interactions within broader social and community contexts, including relationships with other residents, interactions with staff members, organizational leadership, and broader societal attitudes toward race and ethnicity. Exploring the influence of race and ethnicity on RRA through a socioecological lens can therefore help the development of targeted prevention strategies, as effective violence prevention requires integrated strategies that address risk factors at every level rather than focusing solely on individual residents (Pillemer et al., 2012). For example, at the individual level, two residents’ impaired cognitive health, disinhibition, and behavioral disturbance may lead to an acute RE-RRA episode, such as exchanging racial slurs (Ramirez et al., 1998). At the staff level, the lack of training may leave staff unprepared to mitigate conflicts between residents from different racial/ethnic backgrounds. At the facility level, intergroup tension may be exacerbated due to the lack of organizational readiness or capacity to support change toward more inclusive environments (LeBlanc & Kelloway, 2002).

Additionally, we also adopted an event-based perspective to examine how RE-RRA unfolds over time across each socio-ecological level (Wilkinson & Hamerschlag, 2005). The event-based perspective, which has been used in prior elder mistreatment and RRA research, helps explain how and why aggression occurs (Chang et al., 2024; Rosen et al., 2019). This approach distinguishes between predisposing factors, or long-term vulnerabilities that increase the likelihood of aggression, and acute precipitants, which are defined as immediate stressors that trigger an incident in the moment. Differentiating the two may contribute to developing effective strategies for violence prevention, such that primary prevention efforts can focus on reducing predisposing factors, while secondary prevention can address acute precipitants as they arise (Bell & Naugle, 2008).

Finally, insights from stigma-based aggression offer a useful lens for understanding drivers unique to RE-RRA. Stigma-based aggression, which targets individuals because of their race, ethnicity, or other marginalized identities, has been distinguished from general aggression arising from interpersonal conflict without identity-based motivations (Sapouna et al., 2023; Xu et al., 2020). Because the social and cognitive processes differ between the two, risk factors are likely to diverge, with domain-general risk factors that predict aggression broadly, and domain-specific risk factors that uniquely predict stigma-based aggression (Earnshaw et al., 2018). Examples for domain-specific risk factors for stigma-based aggression may include structural/community factors (e.g., social hierarchies, neighborhood conditions), group processes (e.g., intergroup contact, group norms), and stigma-specific mechanisms (e.g., prejudice, discrimination) (Stangl et al., 2019). Effective prevention and response to stigma-based aggression, including RE-RRA, requires tailored strategies that address domain-general and stigma-specific domains.

Using the approach combining the socio-ecological, event-based, and stigma-based aggression perspectives, we sought to: (1) describe the phenomenon of RE-RRA, (2) examine predisposing and precipitating factors unique for RE-RRA, as well as (3) identify factors that are shared between general RRA and RE-RRA.

## Research design and methods

### Study design and setting

We selected a qualitative study design because it allows researchers to characterize RE-RRA from diverse, lived perspectives; it also supports the exploration of interpersonal processes for understudied topics (Maxwell, 2009). We used the Consolidated Criteria for Reporting Qualitative Research (COREQ) as a guideline for reporting results (see Supplementary Material 1) (Tong et al., 2007). We recruited two LTC sites in a large metropolitan area on the U.S. East Coast. Both facilities were large, not-for-profit, chain-affiliated LTCs with long-term care floors, and differed in the racial/ethnic composition of their resident populations to capture variations in context relevant to RE-RRA. LTC site 1 had a mostly non-Hispanic White population (79% non-Hispanic White; 8.5% Black; 7.5% Hispanic), while LTC site 2 was highly racially and ethnically diverse (28% non-Hispanic White; 44.5% Black; 24.8% Hispanic).

Prior to fieldwork, the research team established formal partnerships with each organization’s leadership. The executive teams authorized on-site recruitment and coordination with unit managers. With administrative approval, study staff posted Institutional Review Board (IRB)-approved flyers in staff lounges and other staff-only common areas in the facilities. Participants were recruited via purposive sampling (Campbell et al., 2020), and were eligible if they were direct-care, clinical, or non-clinical staff working on long-term care units who had at least three months of experience in current positions. Prospective participants could choose either a focus group or an in-depth, semi-structured interview, based on their preference and/or work schedule. Participants received a $20 e-gift card upon completion of the interview or focus group. All interviews were audio-recorded and transcribed. Transcripts were deidentified for data analysis. The final sample size was determined inductively by achieving thematic saturation. Through a concurrent coding process, there were no new codes emerged from additional interviews (Sandelowski, 1995).

### Data collection

We elected to conduct both focus groups (FG) and semi-structured in-depth interviews (SI). Researchers have considered the combination of multiple qualitative data collection methods as an advantageous approach to exploring complex interpersonal phenomena, because it allows for leveraging the strengths of each method (Morse, 1999). Focus groups allowed us to capture shared language and interactional dynamics among LTC staff, while semi-structured interviews enabled us to explore more individualized and sensitive experiences in-depth (Lambert & Loiselle, 2008). We developed an interview guide based on existing literature, theoretical frameworks including the socioecological framework, an event-based perspective, and stigma-based aggression, in addition to our team’s content expertise. Key open-ended questions included: (1) Tell me about your experience with RE-RRA. Can you describe an incident that you have witnessed? (2) Why do you think these incidents happen? (3) Is there anything about the residents, staff members, or facility that shape these incidents, or trigger these incidents at the moment? To ensure clarity, we pilot-tested the guide with two certified nursing assistants, two nursing home physicians, and one public health researcher.

Before the interviews, the moderator reviewed the purpose of the study, its research objectives, and briefly introduced the study process. Participants self-reported gender, race/ethnicity, experience in the healthcare field, and current position in the facility. A total of 81 staff members participated. There were nine focus group discussions, each including 3 to 16 participants (mean number = 7.6), in addition to 12 one-on-one semi-structured interview sessions. Interviews lasted on average 36 minutes (range: 23–50 minutes). No participants dropped out. The lead author (E.-S.C., PhD in Social and Behavioral Sciences), trained in qualitative research, conducted all the focus groups and semi-structured interviews. Only the research team and participants were present during the focus groups and interviews. All interviews took place in staff meeting rooms or respite lounges at the facilities and occurred between March and June 2024. This qualitative work is part of a larger, ongoing exploratory sequential mixed-methods project to develop and refine a novel staff-focused educational intervention aimed at preventing and mitigating RRA.

### Data analysis

We employed a thematic qualitative approach to code transcripts by assigning codes to blocks of text that corresponded to the code definitions (Kiger & Varpio, 2020). We used emergent themes from our field notes to develop an initial codebook. Transcripts from both focus groups and semi-structured interviews were coded using a single codebook. Using a multiple-coder practice (Cohen & Crabtree, 2008), the first author and a second coder then dual-coded two transcripts to reassess and refine themes and develop the final codebook. As part of the training process, the two coders iteratively coded three transcripts to establish agreement on coding applications, with consensus recorded and documented in detailed coding memos. Two coders independently coded the remaining 16 transcripts. Themes identified were compared between two coders, and disagreements were clarified by consensus involving a third coder. Direct quotes arising from the discussion are presented to support identified themes. To protect participants’ identities, reported role titles were replaced with broader professional categories in the final data tables (e.g., clinical supervisors or administrative staff). We used memos to organize the analytic process and discussed uncertainties with the broader team to reach consensus. To thoughtfully integrate both sources and support the validity of the findings, we applied an a priori analytic plan based on within-method triangulation, including comparing data across interview methods and cross-verification of findings (Carter et al., 2014). Coding was conducted using Dedoose version 9.0.107 and took place between September 2024 and February 2025.

### Trustworthiness of the study

We ensured the trustworthiness of the study by following the critical evaluation criteria for qualitative research, including the principles of credibility, confirmability, dependability, and transferability (Lincoln, 1985). We strengthened credibility by peer briefing and creating team meeting memos. We also applied the member checking technique, inviting three participants to review the coding themes, findings, and data interpretation (McKim, 2023). We further enhanced confirmability by keeping an audit trail of analytic decisions during weekly research staff/coder meetings. Lastly, we ensured the dependability and transferability of the study findings to other contexts by continuing discussions with providers and researchers (LTC providers, geriatricians, public health researchers) who were not part of the study team.

### Researcher characteristics and positionality

The research team included eight experienced researchers, two research trainees, and two LTC clinical providers. The group reflected diversity in age, sex, and disciplinary training, including gerontology, geriatrics, aging and health disparities, public health, and emergency medicine, as well as clinicians and educators involved in care and staff training in LTC settings. Research team members were well versed in qualitative methodology and elder mistreatment research and shared a socioecological perspective on gerontological inquiry. These diverse backgrounds contributed complementary perspectives to data interpretation.

In terms of positionality, the lead author identified as a female and Asian. Team members identified as Asian, Black, and non-Hispanic White and recognized that their lived experiences and viewpoints could influence data interpretation. Team members’ collective expertise in race/ethnicity research and health disparities helped shape our analytic lens. Throughout iterative team discussions, members drew on cultural knowledge while explicitly examining potential biases. This reflexive process supported the refinement of key themes and informed the language used to present our findings.

### Ethics

The Institutional Review Board at Weill Cornell Medical College provided ethical approval for this study (#23-12026873). Each participant provided verbal informed consent before the start of the discussion. Funding was provided by the National Institute on Aging, which played no role in the collection, interpretation, or presentation of the data.

## Results

### Participant characteristics

A total of 81 LTC staff members took part in the study, representing both clinical (81.5%) and non-clinical (18.5%) roles (Table 1). Participants were primarily female (78.3%) and racially/ethnically diverse, with 43.2% identifying as Black, 25.9% identifying as White, 17.3% identifying as Hispanic, and 8.6% identifying as Asian. Staff had an average of 12.6 years of experience working in LTC settings.

**Table 1:** Characteristics of staff participants (*N* = 81).

Female, *n* (%)	59 (78.3)
**Race/ethnicity**	
White, non-Hispanic	21 (25.9)
Black	35 (43.2)
Hispanic	14 (17.3)
Asian	7 (8.6)
Other	4 (4.9)
**Role in practice, *n* (%)**	
Clinical staff	66 (81.5)
Certified nursing assistant (CNA)	18 (22.2)
Registered nurse (RN)	4 (5.0)
Nurse practitioner (NP)	3 (3.7)
Licensed practice nurse (LPN)	6 (7.4)
Clinical supervisor	4 (5.0)
Physician	7 (8.6)
Clinical assistant	3 (3.7)
Physical therapist	4 (4.9)
Occupational therapist	7 (8.6)
Recreational therapist	4 (4.9)
Social worker	3 (3.7)
Dietitian	3 (3.7)
Non-clinical staff	15 (18.5)
Administrative staff	10 (12.3)
Housekeeping	5 (6.2)
Years in practice, M + SD	12.6 + 9.5
**Interview mode, *n* (%)**	
Semi-structure interview	12 (14.8)
Focus group	69 (85.2)

### Staff perception of race/ethnicity-related RRA

Participants used a variety of colloquial terms to describe incidents of RE-RRA. These terms included: “racial conflict”, “racial altercations”, “racial comments”, “race to race”, “a racial thing”, or “racialized interaction”. These intuitive descriptors may illustrate how participants observed RE-RRA in LTC social encounters, even if these incidents were not formally labeled. Indeed, participants described RE-RRA as a recurring feature of interpersonal dynamics, reporting that aggression “normally happened in different backgrounds” when residents from different racial/ethnic groups interacted. As a participant reported, “I feel a lot of [aggression] starts from different ethnic backgrounds, different accents. I feel like racial comments start when, you know, residents get aggravated” (FG 5, #1, certified nursing assistant).

Additionally, participants also shared nuanced views about the perceived salience of RE-RRA. Several participants described RE-RRA as less prominent than other forms of resident conflict stemming from background and lifestyles differences in sex or gender, personality, or socioeconomic statuses. Others noted RE-RRA as context-dependent, such that it does not uniformly take place across all units. Rather, its occurrences hinged upon specific resident dyads and pairings. For example, a participant compared the prevalence of RE-RRA in long-term versus short-term units: “People who are here for short term are more focused on their therapy and getting stronger and getting out. I don’t think they are looking forward to having an interaction [with others]. They are more respectful towards each other” (FG3, #3, occupational therapist).

Participants further noted that the full extent of RE-RRA may be unnoticed or unreported because these incidents have become normalized within the care environment. As a participant explained, “I mean, I don’t see it so much here. But again, it probably does occur more than I think… residents may say otherwise, you know, maybe [staff] are just not so attuned to it” (SI10, physical therapist). Others noted the unreported nature of RE-RRA. A participant shared: “It’s kind of papered over. You know, no one really wants to explore in depth, because it’s a huge can of worms” (SI12, registered nurse). Notably, repeated exposure to RE-RRA appeared to contribute to this desensitization and subsequent normalization. A participant reported being desensitized to RE-RRA unless the incidents are overt: “We are just used to it […], so you say that it doesn’t affect you anymore. Unless it’s like blatant, you know?” (FG5, #4, certified nursing assistant).

### Types of race/ethnicity-related RRA

A key finding of the study was the identification of distinct types of RE-RRA, each with different characteristics and potential intervention implications. Five overarching categories were identified. Within each broad category, we provide several examples.

#### Physical violence with racial/ethnic overtones

Likely to cause immediate harm and injury, participants reported physical acts of hitting, pushing, grabbing, kicking, or striking with objects that are accompanied by racialized language or directed toward another resident from a different race/ethnicity. As a participant reported, “they [residents] actually became physically abusive—so frustrated that they would use whatever was nearby to hit the other person.” (FG1, #4, nurse practitioner).

#### Verbal harassment with explicit racial/ethnic slurs

Participants reported behaviors of overt derogatory statements that denigrate residents’ racial/ethnic backgrounds, including racial/ethnic slurs, exchange of verbal insults, or profanity. As a participant described, residents may say “really nasty and vulgar things” to others from different races/ethnicities. A commonly cited example is racial/ethnic slurs: “Resident is saying the N word [to another resident]. That’s how he feels” (FG6, #6, housekeeping).

#### Racial/ethnic stereotyping

Participants reported that residents may make snap judgments or generalized assumptions about others from different racial or ethnic backgrounds, which can make the target recipient feel excluded and create an unwelcoming social environment. For example, invalidating assumptions that “go by someone’s accent”.

#### Racial/ethnic microaggressions

This form consists of subtle, indirect, or sometimes ambiguous comments and behavioral exchanges between residents from different racial or ethnic backgrounds. Even if they may not be explicitly hostile, the receiving resident(s) may perceive it as hurtful. For example, a participant stated that there appeared to be many types of racial altercations, some forms were said to be hurtful, while others were not necessarily intended to be hateful: “Sometimes it may be a joke, and then a third person may take offense” (FG8, #4, licensed practical nurse).

#### Social exclusion and avoidance behaviors

This form involves residents who deliberately refuse to engage with or distance themselves from residents from different racial/ethnic backgrounds. Examples included refusing to sit together, join programs, or share spaces. A participant noted, “It can be that someone who does not want to join [group] activities to avoid interacting with another ethnic group” (FG1, #4, nurse practitioner).

### Predisposing factors and acute precipitants for RE-RRA

Participants identified a constellation of factors that predispose or precipitate RE-RRA. While many contributors are shared between general RRA and RE-RRA, several factors are uniquely related to race and ethnicity. We present findings below by socioecological level (resident, staff, and facility) and factor type (unique predisposing factors and acute precipitants for RRA versus shared predisposing factors and acute precipitants with general RRA) (Table 2).

**Table 2:** Predisposing factors and acute precipitants of race/ethnicity-related resident-to-resident aggression (RE-RRA).

Factor type	Themes	Representative quotes
**RESIDENT LEVEL**		
**PREDISPOSING FACTORS**		
Factors unique to RE-RRA	Explicit racial bias	“Sometimes one says that I don’t like Spanish, I don’t like Black. I don’t like this one. Sometimes they’d be straight-up racist.” (FG7, #2, certified nursing assistant) “It’s like, one resident said, ‘Don’t touch me, Blackie,’ to another resident in the room.” (SI#9, social worker)
	Implicit racial bias	“You know, we like to say sometimes, ‘Oh, well, they have dementia.’ But maybe it’s because they just have an underlying bias anyway.” (SI#11, physical therapist) “Residents can feel tension over ethnic cues, and these cues change how they respond to each other.” (FG8, #5, social worker)
	Segregated upbringing	“I think you deal with people born in certain time periods. There’s some inherent racism. If they grew up during segregation, some of them hold on to those beliefs.” (SI#9, social worker) “If you were young, and you were raised in a racial [setting], where you didn’t like Black people or you didn’t like White people, you might react in a behavior that’s just not appropriate, because that’s what you remember from when you were a kid.” (FG4, #1, certified nursing assistant) “[RE-RRA is], because, I think, racial behaviors for this older generation are ingrained.” (FG6, #4, certified nursing assistant)
Factors shared between general RRA and RE-RRA	Dementia/cognitive impairment	“Dementia, the patients with dementia…. They can get in conflict with anybody, color, race, creed does not matter.” (FG9, #1, certified nursing assistant) “A significant number of residents are cognitively impaired. So, you know, someone’s screaming ‘ow, ow’; and you know, the normal reaction for someone with dementia is just like, ‘shut up’.” (FG2, #11, administrative staff) “It is because some residents are wandering. Again, that’s a component of dementia.” (SI#11, physical therapist)
	Social adjustment challenges	“I think it has to do a lot with them [residents] not being at home anymore. Now [they are with] different staff, different people, they’re not in a room by themselves. So, you know, [residents] get aggravated because they’re not able to just walk out of the facility and do what they want, at any time. So, you know, that plays a role in how they behave as well.” (FG5, #4, certified nursing assistant) “They are angry because they want their life back. I noticed that they were angry because they want to be like us and they can’t anymore.” (FG4, #1, certified nursing assistant)
	Psychiatric comorbidities	“In our population, we have a huge subset with psychiatric illnesses, behaviors, and a lack of impulse control. So, we see a lot of interactions, negative interactions, secondary to inability to self-contain themselves… and that actually just leads to repetition of negative interactions, because some of those residents don’t forget.” (FG2, #12, clinical supervisor) “Some people have like bipolar or are on different kinds of medications, and they have behavioral issues.” (SI#15, registered nurse)
	Pain/discomfort	“A lot of times, residents have medical conditions, [and] they’re in pain. When they’re in pain, they lash out.” (SI#4, social worker) “They are frail, they are bed-bound, and they’re frustrated that they can’t walk into anything, and they just want to yell.” (SI#9, social worker)
	Medication issues	“Sundowning is a factor. Changing medication is a factor.” (SI#3, administrative staff) “Some of them are on different kinds of medications, and they have behavioral issues. So, that’s what happens.” (SI#16, registered nurse)
**ACUTE PRECIPITANTS**		
Factors unique to RE-RRA	Racial/ethnic slurs	“I think sometimes when people are [cognitive impaired], [residents] say racial comments that maybe, you know, they wouldn’t say when they are not impaired. But that does not take away from the effect.” (SI#10, registered nurse) “You’ll see two residents with dementia. They’re hitting each other, they’re saying racial slurs. They’ll always remember that ‘I’m better than you.’” (FG2, #9, clinical supervisor)
	Interracial/ethnic communication misinterpretation	“Language and communication are big issues. Certain cues are different across different cultures. Even tones. Sometimes you have to speak louder in certain cultures, or else they think you’re being disrespectful. But if you’re too loud, then they think you are being too rude.” (FG7, #5, dietitian) “Some people don’t like eye contact. Some people do. Like, a sign of showing eye contact could be respectful to some cultures, or it could be disrespectful to someone in other cultures.” (FG7, #2, certified nursing assistant)
	Perceived racial prejudice in staff treatment	“I do believe that when we come from different cultural backgrounds—as someone who doesn’t speak the native language—you do feel some way about a resident who does speak English and was born in this country. That can cause a negative interaction, because it’s almost like this idea of ‘survival of the fittest.’ You feel like you can’t survive, and the other person is always ‘eating before you’ because they speak the language. I’ve seen that here, and it comes up a lot in conversations with residents: ‘Well, I don’t like this person because he speaks this language, or he was born here, and I believe he gets treated better.’” (FG2, #2, social worker)
Factors shared between general RRA and RE-RRA	Behavioral disturbances	“The patients with dementia don’t have a filter. Filter gets erased when [residents have] dementia. So, um, even with us working with them, they have no filter. They say things that are very inappropriate [and trigger aggression].” (FG6, #4, certified nursing assistant) “When you have dementia, or other certain diseases, you’re uninhibited. So, you just say what’s on your mind.” (SI#8, certified nursing assistant)
	Competition for staff attention	“Okay, let’s say there was a resident who was one-to-one on one of those floors. Then another resident that was in the same room was like, ‘How come you get all the attention and I don’t any’. So it’s kind of like a lot of jealousy without finding out the reason.” (FG6, #3, registered nurse)
	Perceived unfairness in care	“Sometimes residents feel that we’re showing favoritism to someone of higher status who has higher insurance, as if some residents are treated as VIPs. That often starts some negative interaction between them.” (FG2, #1, social worker)
**STAFF LEVEL**		
**PREDISPOSING FACTORS**		
Factors unique to RE-RRA	Explicit racial bias	“We all come with our own biases, even if we shouldn’t. I think that, you know, if I’m Jewish, and I see someone say an anti-Jewish comment to another resident, I would be more protective in that situation. Not that we should do that, because it shouldn’t matter. Any racial or discriminatory incident should be treated in the same fashion. But I think we all have our own biases. So it’s hard to completely disassociate.” (FG2, #8, clinical supervisor)
	Implicit racial bias	“When dealing with residents with altered mental status, sometimes staff go by someone’s accent or background to anticipate behavior, even if they don’t realize it.” (SI#3, administrative staff)
	Inadequate training in cultural sensitivity	“I think is really important to know how to speak to people. It is really an art to speak to someone. You have to know exactly how to choose your words wisely. So people don’t get offended because they are from different [backgrounds].” (FG7, #2, certified nursing assistant) “Having some knowledge about cultures that are not your own would help eliminate bias and certainly be beneficial [to address RE-RRA].” (FG5, #2, licensed practical nurse)
Factors shared between general RRA and RE-RRA	Tolerance of violence	“We’re just used to it. It’s like, you see it to the point where sometimes it’s hard to brush it off. So, you say that it doesn’t affect you anymore. Unless it’s like blatant, you know? (FG5, #4, certified nursing assistant)
	Inadequate training in managing resident aggression	“It’s not in the curriculum. I think it could be beneficial just to learn how to kind of control that situation. I feel like we kind of redirect, but not necessarily, in the moment, know what to do.” (FG6, #4, certified nursing assistant)
	Inadequate training in dementia care	“We have seen a decrease in our resident-to-resident [negative] interactions over the past couple of years. But now that we’re increasing our dementia population, this is a perfect time for additional dementia training to prevent [negative] interactions.” (FG2, #10, administrative staff)
	Poor staff-resident rapport	“Sometimes residents have difficult experiences, and conflicts happen more easily because of these experiences. Sometimes that includes frustration with staff.” (SI#5, certified nursing assistant)
**ACUTE PRECIPITANTS**		
Factors unique to RE-RRA	Challenges in impartiality/mitigating bias in intervention	“I always feel not both [i.e., aggressor and victim] are satisfied with the intervention, you know, someone always gets the shorter end of the stick. Sometimes we might favor one [over the other] if we understand them more. So what do we do with the one who didn’t feel like they were understood… We just hope they don’t loath us later.” (FG8, #5, dietitian)
	Interracial/ethnic communication misinterpretation	“It’s really just the inability to communicate—when different ways of communication are mixed, with one person unable to communicate because of dementia and another speaking a different language.” (FG2, #14, administrative staff)
Factors shared between general RRA and RE-RRA	Delayed or inactive intervention for resident interpersonal aggression	“I tried [intervention] before and you can get hurt. I try not to get involved in it. Because I’ve been hit.” (SI#1, certified nursing assistant)
**FACILITY LEVEL**		
**PREDISPOSING FACTORS**		
Factors unique to RE-RRA	Social hierarchies	“I feel like a big problem is White people. They don’t really understand the oppression of other people. White people have privilege …. They don’t get the negative, vulgar, disrespectful comments on the street, and they don’t understand how that affects other cultures. Even among the residents, they’re quick to judge [each other]—just by looking at their skin color.” (FG5, #1, certified nursing assistant)
	High racial/ethnic heterogeneity	“In a place with a lot of different races and backgrounds, sometimes residents just don’t understand each other, and small things blow up faster than you’d expect.” (SI#3, administrative staff) “I feel like it’s not the people themselves, it’s the differences. The more different they are, the more chances for misunderstandings, and then it can turn into aggression.” (SI#1, certified nursing assistant)
	Lack of racial/ethnic heterogeneity	“Based on my experience, it’s obviously going to happen in places where they have more Caucasians, and few minorities.” (SI#5, certified nursing assistant) “[Where I used to work], there was no diversity of staff. There was no diversity even among patients. So yes, I did witness some aggression that appeared to be directed toward individuals of African American, or even of Hispanic backgrounds, because they were the only people of color in those settings. But I don’t see that here [in the current care environment], because I think here people are more sensitized to seeing different people.” (FG1, #10, clinical supervisor)
	Lack of intergroup activities	“On one of the units we have a very large, um, Spanish speaking population, they’re either Spanish speaking only or Spanish speaking as primary language. So, in recreation, we fostered a Latin club, so they had the opportunity to speak with Spanish peers, so they don’t feel isolated that they don’t speak English or another language, to help them bond and not feel targeted.” (FG2, #8, recreational therapist)
Factors shared between general RRA and RE-RRA	Chronic understaffing	“Every department needs more staff. And that’s what I think. If you have enough staff, then again, with education, you know, you can better train the staff.” (FG6, #6, housekeeping)
	Financing limitation	“It has to do with the dynamics of healthcare and the budget and whatever they can afford to have these things.” (FG6, #1, certified nursing assistant) “It can be that mental health facilities are closing down, and then you put them in a facility with vulnerable elderly people, and it’s just… Oh, my gosh. You know.” (FG6, #2, physical therapist)
	Increasing number of residents with dementia	“When you mix a large community of dementia patients [with patients that don’t have dementia] then you get those issues with roaming and everything else.” (FG1, #5, physician)
**ACUTE PRECIPITANTS**		
Factors unique to RE-RRA	Close/forced interactions of residents of different races/ethnicities	“My perspective is that forced interactions between residents from different races—when they’re taken into programming—lead to a kind of forced interaction that doesn’t happen intrinsically or with autonomy, because it involves strangers being placed into an environment they’re not familiar with.” (FG2, #12, clinical supervisor)
Factors shared between general RRA and RE-RRA	Shared living arrangement	“You see it is not easy, living with people you don’t know.” (SI#2, certified nursing assistant) “[One] thing also with [this nursing home is that] it is largely private rooms. So, for a patient, you know, you might not like what you see outside, but you can go back to your room and have your own space. And I see that conflicts more often in other nursing homes where [residents have to share rooms].” (FG1, #5, physician)
	Lifestyle/behavioral norms clashing	“When I’m admitting a patient who’s used to street culture into a facility regulated by external bodies, you’re expected to behave in a certain way, and a resident who’s been in the building and has acclimated to what the internal community should look like. You’re going to have resident-to-resident [aggression] when somebody is operating outside of what that external street culture would look like.” (FG2, #11, administrative staff)

### Resident-level factors

#### Unique predisposing factors for RE-RRA

Participants described several predisposing factors rooted in race-related attitudes and histories. Explicit racial bias (e.g., “straight-up racist”), implicit racial bias (e.g., “tension over ethnic cues”), and segregated upbringing were reported as factors shaping residents’ perceptions and responses to one another. As a participant noted, “If you were raised in a racial [setting]… you might react in a behavior that’s just not appropriate, because that’s what you remember from when you were a kid” (FG4, #1, certified nursing assistant).

#### Shared predisposing factors with general RRA

Participants described several clinical characteristics that created a baseline vulnerability for RRA, including dementia, psychiatric comorbidities, pain, and medication changes. Dementia was the most commonly reported factor, given its effect on cognitive function that lowered residents’ threshold for conflict, such that “they [residents] can get in conflict with anybody, color, race, creed does not matter” (FG9, #1, certified nursing assistant). Social adjustment difficulties were also noted, as residents struggled with the loss of autonomy and difficulties adjusting to LTC social dynamics: “they [residents] get aggravated because they’re not able to just walk out of the facility and do what they want at any time” (FG5, #5, certified nursing assistant).

#### Unique acute precipitants to RE-RRA

Participants noted the use of racial/ethnic slurs, interracial/ethnic communication misinterpretation, and perceived racial/ethnic prejudice in staff treatment as the main situation triggers. Though participants commonly attributed residents’ use of racial or ethnic slurs to cognitive impairment or disinhibition, they emphasized that such explanations did not diminish the harm experienced. As a registered nurse shared: “They [residents] say racial comments that maybe, you know, they wouldn’t say when they are not impaired. But that does not take away from the effect.” (SI10, registered nurse).

#### Shared acute precipitants with general RRA

Participants reported common situational triggers, including behavioral disinhibition and neuropsychiatric symptoms associated with dementia (e.g., “filters get erased when [residents] have dementia”; “wandering behavior”), competition for staff attention (e.g., “How come you get all the attention and I don’t”), and perceived unfairness in care, whereby certain residents were viewed as “VIPs”, provoking negative perception and resentment from peers.

### Staff-level factors

#### Unique predisposing factors for RE-RRA

Participants reflected that their own explicit and implicit racial biases may shape how they react to RE-RRA. A participant reflected that “sometimes staff go by someone’s accent or background to anticipate behavior, even if they don’t realize it”, suggesting that such biases may influence perceptions of fairness and indirectly affect interactions among residents of different racial or ethnic backgrounds interact. Others highlighted the lack of training in cultural sensitivity and awareness: “It is really an art to speak to someone. You have to know exactly how to choose your words wisely. So, people don’t get offended because they are from different [backgrounds]” (FG7, #2, certified nursing assistant).

#### Shared predisposing factors with general RRA

Participants described several staff-level factors that affect their confidence and capacity to effectively respond to RRA, therefore contributing to an environment that makes aggression more probable. Participants reported limited training in aggression management and specialized dementia care as skills that were “not in the curriculum”. Finally, some noted that poor staff–resident rapport can increase risk of conflict: “Sometimes residents have difficult experiences, and conflicts happen more easily because of these experiences [including] frustration with staff” (SI5, certified nursing assistant).

#### Unique acute precipitants for RE-RRA

Participants reported that the challenge of maintaining impartiality when managing residents from different racial/ethnic backgrounds can act as a stimulus for RE-RRA: “Sometimes we might favor one [over the other] if we understand them more. So, what do we do with the one who didn’t feel like they were understood… We just hope they don’t loathe us later.” (FG8, #5, dietitian). The struggle to remain neutral could inadvertently amplify residents’ sense of inequity and trigger RE-RRA.

#### Shared acute precipitants with general RRA

Participants noted that delayed or inactive interventions, often due to fear of injury or uncertainty about proper intervention, could trigger RRA. A participant recalled, “I tried [intervention] before and you can get hurt” (SI1, certified nursing assistant). Such hesitation might allow manageable tension to escalate into overt aggression.

### Facility-level factors

#### Unique predisposing factors for RE-RRA

Participants contextualized RE-RRA within broader pre-existing social hierarchies. For example, participants noted that residents’ interactions often reflected racial advantages embedded in broader social norms: “[Residents] are quick to judge [each other]—just by looking at their skin color” (FG5, #1, certified nursing assistant), and that White residents may be viewed as less likely to experience adverse experiences in LTC compared to residents of color. Interestingly, both highly racially/ethnically diverse and homogeneous groups were described as at-risk conditions. In highly diverse LTC settings, episodes can occur frequently (e.g., “small things blow up faster than you’d expect”). Yet, environments where the majority are White residents may also increase risk for residents from minority backgrounds (e.g., “because they were the only people of color”). The lack of inclusive intergroup activities was viewed as compounding these risks.

#### Shared predisposing factors with general RRA

Chronic understaffing was cited as heightening the risk of RRA. As a participant remarked, “needs more staff. And that’s what I think. If you have enough staff, then again, with education, you know, you can better train the staff”. (FG6, #6, housekeeping). Participants also pointed to other system-level pressures, such as broader healthcare financing limitations and the increasing number of residents with dementia, which heightened the complexity of care and frequency of resident interactions: “When you mix a large community of dementia patients [with patients that don’t have dementia] then you get those issues with roaming and everything else” (FG1, #5, physician).

#### Unique acute precipitants for RE-RRA

A recurring theme was structured activities that forced social mixing across racial or cultural lines. As a participant noted, “It involves strangers being placed into an environment they’re not familiar with” (FG2, #12, clinical supervisor). These encounters may intensify existing cross-group tension.

#### Shared acute precipitants with general RRA

Participants described several acute triggers embedded in the communal structure and environmental design of LTC, including conflicts arising from daily shared living challenges (e.g., “not easy living with people you don’t know” and the lack of private rooms), and clashes occurring because of inter-group differences (e.g., “[a resident who] has acclimated to [the] internal community” versus “[somebody operating out of] the external street culture”).

### Summarizing findings into conceptual framework

We summarize these findings into a figure (Figure 1) that illustrates predisposing and precipitating factors for RE-RRA at the resident, staff, and facility levels. This may serve as a conceptual framework to inform future research and guide interventions.

**Figure 1:**
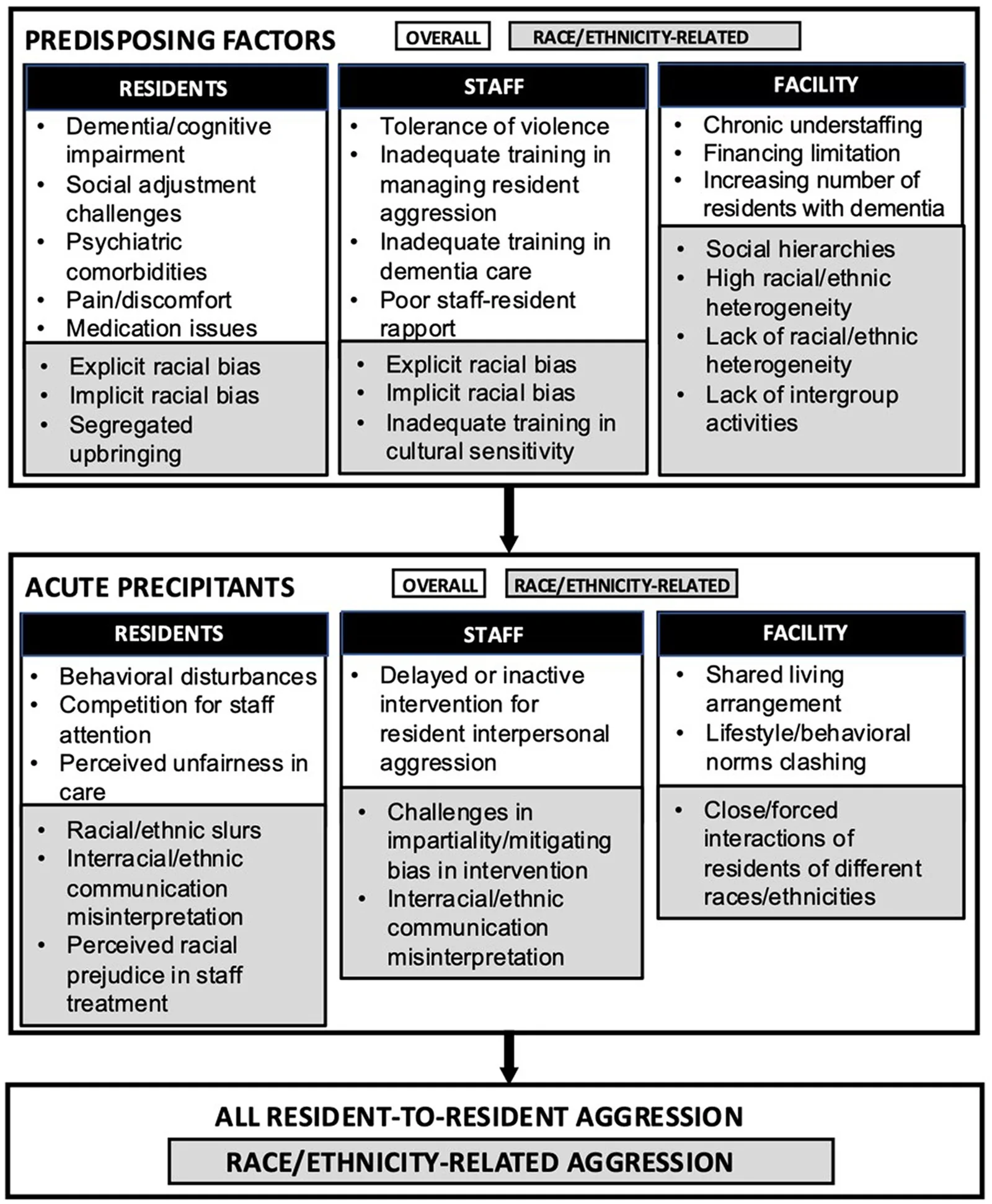
An illustrative figure highlighting predisposing factors and acute precipitants of race/ethnicity-related resident-to-resident aggression.

## Discussion

This study provides one of the first in-depth examinations of RE-RRA in LTC settings. Our findings suggest that RE-RRA is likely common but often under-detected, despite its wide-ranging manifestations that spanned from explicit physical violence to implicit racial micro-aggression. By applying a socioecological framework, an event-based perspective, and principles of stigma-based aggression, we identified a broad array of predisposing factors and acute precipitants that may uniquely drive RE-RRA incidents, as well as those shared with general RRA across nested multiple socio-ecological levels. These findings underscore the importance of considering RE-RRA as a distinct type of RRA, and carry direct implications for the design and implementation of prevention and intervention strategies.

To our knowledge, this study represents the first effort to characterize a broad spectrum of RE-RRA incidents. Our findings suggest that RE-RRA are not only confined to overt hostility, such as explicit racial/ethnic conflicts (Chang et al., 2024). Instead, RE-RRA may manifest through a constellation of different behavioral patterns and expressions that varied in incident intensity and visibility. Notably, despite the fact that RE-RRA may occur in staff’s day-to-day observations, episodes are likely to be overlooked due to limitations in awareness, training, or competing care demands. As a result, these incidents may cause serious harm to the involved residents and the staff and create an unwelcoming social environment for everyone.

Our findings advance knowledge in RRA by identifying new domains of modifiable risk factors specific to race and ethnicity. Prior research in RRA has primarily focused on residents’ health and clinical characteristics as well as facility-level risk factors, as key predictors of aggression (Ferrah et al., 2015; McDonald et al., 2015; Pillemer et al., 2012; Schiamberg et al., 2015). Our findings indicated that race/ethnicity of involved residents, dyads, staff members, and the surrounding workforce dynamics may all create an environment in which RE-RRA can emerge or escalate. Most notably, staff participants described how their own implicit and explicit biases, as well as residents’ perception of bias in staff actions, may work in tandem to influence the emergence of RE-RRA. Broader structural challenges, including chronic staffing shortages, high turnover rates, and lack of training in RE-RRA may further exacerbate these risks. Recognizing staff bias as part of the ecology of RE-RRA is critical, as these perceptions may be modifiable (Devine et al., 2012), and can shape which incidents are addressed promptly or minimized. Indeed, existing research in healthcare suggests that, relative to individual and community/facility-level factors, staff-level factors may be the most amenable to change and may be targeted first (Hagiwara et al., 2019).

Interestingly, staff also recognized that residents’ perception of staff bias may play a role in RE-RRA, indicating that staff-resident interaction should be considered as part of the broader interpersonal dynamics within the care environment. This is consistent with prior findings that aggressive behaviors may be a learned behavior contributing to a climate of violence in a care environment (Schiamberg et al., 2015). Perceived staff bias may also trigger residents’ violent response to aggression based on a lifetime of exposure to racism. As such, different forms of interpersonal aggression occurring in LTC may be closely intertwined and warrant further investigation.

Another contribution of our research is distinguishing and categorizing factors contributing to RRA as predisposing and precipitating. This approach, used previously in other areas of elder mistreatment research (Rosen et al., 2019, 2024), provides a helpful framework for targeted prevention. For example, preventing RE-RRA among residents with dementia may require addressing predisposing risks through tailored dementia care education and strengthened organizational support, while equipping staff with real-time cross-cultural communication skills to manage acute triggers as they arise.

Our findings also suggest that new instruments are needed to detect and track RE-RRA specifically. Currently, we are aware of only one RRA measurement tool that includes a racial or ethnic component. This tool is limited to asking about the explicit use of racial or ethnic slurs (Ramirez et al., 2013), and therefore, does not identify all RRA in which race/ethnicity plays a role. Improving measures to detect RE-RRA may help ensure that these incidents are recognized and addressed.

This study has several limitations. First, we recruited from two LTC sites in a large metropolitan area. Although these facilities differed in resident demographics, they may not fully capture the range of organizational structures and cultural contexts across broader LTC settings. Future studies should include a larger and more diverse sample of facilities across multiple regions to improve the generalizability of findings. Second, our data relied on staff perspectives rather than direct reports from residents and families. Including resident and family members’ narratives in future work would provide a more comprehensive understanding of how RE-RRA is experienced and interpreted by those most directly affected. Third, as with many qualitative studies, we aimed to describe the dynamic processes involved in RE-RRA. Although our findings differentiated predisposing vulnerabilities and acute triggers that emerged from data, these factors may overlap, and pathways should be further tested in future research using mixed-methods or longitudinal study designs. Despite these limitations, this study offers a preliminary conceptual map for future inquiry to guide much-needed RE-RRA prevention and intervention strategies.

Our results highlight several areas for improvement in practice across different levels of care. At the frontline level, direct care workers have expressed a strong need for practical tools and training to navigate racial and ethnic dynamics in resident care and social relations. Developing and providing LTC staff with evidence-based training in interactive communicative tools, stereotype recognition and management, and structured de-escalation skills, particularly in the context of dementia care, may help better equip staff to manage these situations effectively (Rosen et al., 2016).

At the care environment level, consistent with prior findings (Hollowood et al., 2026; Xiao et al., 2022), we found that thoughtful, culturally sensitive social activities are needed to avoid discomfort across social groups. To address this, LTC leadership may focus on creating and incorporating initiatives and strategic plans that promote positive social connections into all internal policies, programs, and evaluations (Chapman et al., 2024). Additionally, to improve LTC leadership accountability, from an operational standpoint, our findings also underscore the importance of embedding RE-RRA within existing clinical governance structures, including incident reporting systems, quality assurance and performance improvement processes, and staff education.

Finally, addressing the structural determinants for RE-RRA identified by staff participants in this study will likely require broader policy and workforce reforms that meet the needs of all LTC residents and staff members. Broader societal and policy contexts may influence the resources and capacities of LTC environments and leadership to address RE-RRA. These efforts may include adopting culture change transformation, increasing reimbursement to improve residents’ care and social experiences, and enhancing caregiver training in the LTC workforce (McMullen & Travers, 2023; Travers et al., 2023). Preventing and mitigating RE-RRA will require active leadership engagement, including integration into quality monitoring, continued staff in-service training, and clinical oversight structures. Addressing RE-RRA as a patient safety and quality-of-care priority may help strengthen organizational readiness and sustain these efforts despite changes in the system-level social and policy factors, ultimately improving the care environment for all.

## Conclusion

RE-RRA is a distinct and common type of aggression between residents in LTC settings, with unique predisposing and precipitating factors. Further research is needed to improve understanding of the phenomenon and develop intervention and prevention strategies to enhance the quality of care for an increasingly diverse LTC population.

## Supplementary Material

gnag209_Supplementary_Data

## Data Availability

The data from this study are available from the authors upon reasonable request. This study was not preregistered.
